# Advanced and Metastatic Non-Melanoma Skin Cancer: Epidemiology, Risk Factors, Clinical Features, and Treatment Options

**DOI:** 10.3390/biomedicines12071448

**Published:** 2024-06-28

**Authors:** Zoe Gabrielle Attal, Walid Shalata, Arina Soklakova, Lena Tourkey, Sondos Shalata, Omar Abu Saleh, Fahed Abu Salamah, Ibrahim Alatawneh, Alexander Yakobson

**Affiliations:** 1 Medical School for International Health, Faculty of Health Sciences, Ben Gurion University of the Negev, Beer Sheva 84105, Israel; 2 The Legacy Heritage Center, Dr Larry Norton Institute, Soroka Medical Center, Ben Gurion University, Beer Sheva 84105, Israel; 3 Nutrition Unit, Galilee Medical Center, Nahariya 22000, Israel; 4 Department of Dermatology and Venereology, The Emek Medical Centre, Afula 18341, Israel; 5 Department of Dermatology, Soroka Medical Center and Ben Gurion University, Beer Sheva 84105, Israel

**Keywords:** non-melanoma skin cancers, basal cell carcinoma, cutaneous squamous cell carcinoma, hedgehog, sonidegib, vismodegib, cemiplimab, pembrolizumab, nivolumab

## Abstract

Non-melanoma skin cancers (NMSC) form the majority of skin cancers, with basal cell carcinoma (BCC) being the most common and cutaneous squamous cell carcinoma (cSCC) being second. Prolonged ultraviolet (UV) exposure, aging, male gender, and immunosuppression represent most of the causes of this category of diseases. BCCs and cSCCs both include different types of skin cancers, such as nodular or morpheaform BCC or flat cSCC. Locally advanced and metastatic NMSCs cannot be treated surgically; thus, systemic therapy (TKI and Immunotherapy) is needed. Interestingly, NMSCs are frequently linked to abnormal Hedgehog (HH) signaling which most systemic immunotherapies for these cancers are based upon. Of note, the first line therapies of BCC, sonidegib and vismodegib, are HH inhibitors. Programmed death receptor 1 antibody (PD-1) inhibitors such as cemiplimab, pembrolizumab, and nivolumab have been approved for the treatment of cSCC. Thus, this paper reviews the epidemiology, risk factors, clinical features, and treatment options for both BCC and cSCC.

## 1. Introduction

Due to climate variations, long-term sunlight exposure, and low socioeconomic status, there has been a noticeable global uptick in skin cancer incidence [1,2,3,4]. Recently, it represented around 30% of all cancer diagnoses [5]. The market size of the skin cancer treatment market was valued at USD 7.2 billion in 2021 and is estimated to double by 2031 to around USD 14.5 billion. The sheer size of this global market emphasizes the need for research surrounding skin cancer [6].

Non-melanoma skin cancer (NMSC) is the most frequent type of skin cancer. These include the cancers basal cell carcinoma (BCC) and cutaneous squamous cell carcinoma (cSCC) [7]. However, while BCC represents 75–80% of NMSC, cSCC represents around 15–20%. The incidence of NMSC has steadily increased by around 3% a year for BCC. This review will focus on the latter two NMSCs. NMSCs develop from epidermal cells, and exposure to ultraviolet (UV) radiation can lead to the proliferation of malignant progenitor cells [8]. UVB exposure particularly leads to instability within the DNA of keratinocytes, thus affecting replication. Nonetheless, BCCs are multifactorial and have an array of risk factors that contribute to their development [9]. Additional risk factors comprise specific genetic disorders, fair skin complexion, advanced age, and previous exposure to ionizing radiation [10].

This review will concentrate on advanced and metastatic non-melanoma skin cancers, specifically BCC and cSCC. We will explore various aspects such as epidemiology, risk factors, clinical features, and treatment options (including immunotherapy and the hedgehog pathway) for each diagnosis.

## 2. Materials and Methods

Numerous searches were conducted in PubMed and ClinicalTrials.gov, spanning from their inception to April 2024, aiming to identify clinical trials encompassing locally advanced and metastatic treatments involving Immune Checkpoint Inhibitors (ICIs) and hedgehog inhibitors in non-melanoma skin cancer.

Search terms comprised “non-melanoma skin cancer”, “non-melanoma skin cancer diagnosis”, “non-melanoma skin cancer treatment”, “locally advanced non-melanoma skin cancer”, “metastatic non-melanoma skin cancer”, “ICI’s in non-melanoma skin cancer”, “hedgehog”, “hedgehog signalling”, “hedgehog pathway”, “hedgehog in non-melanoma skin cancer”, “vismodegib”, “sonidegib”, “cemipilimab”, “pembrolizumab”, and “nivolumab”.

Our review encompasses all completed trials for which results are available. The selection of ongoing trials was based on their phase and the treatment administered. All known phase 1, 2, and 3 trials were included.

## 3. Basal Cell Carcinoma

### 3.1. Epidemiology 

Overall, NMSCs occur in around 2 million people globally each year. Their prevalence varies based on the population discussed. BCC is much higher in white populations (75–80% of skin cancers) when compared to darker skin tones (20–30%) [10,11,12,13,14]. While rarely fatal, BCC is potentially disfiguring and harmful to local tissues, making its increase in incidence (3% yearly) problematic. In the odd case of metastasis, BCCs typically spread to local lymph nodes, bones, lungs, and skin [13,14,15]. 

### 3.2. Risk Factors 

Outside of sun exposure, a history of skin cancer (particularly cSCC or previous BCC), age over 50, fair skin, and male gender, for example, are all considerable risk factors for BCC [13]. As male sex and age are risk factors, elderly males are typically at increased risk, further so if they present with fair skin, light eyes, and light hair. Interestingly, other factors include exposure to certain medications (e.g., Hydrochlorothiazide), arsenic, or immune suppression [14]. Additionally, genetics plays a part in the development of BCC. Inherited syndromes particularly increase the risk of BCC. These include basal cell nevus syndrome (also called Gorlin syndrome), Xeroderma pigmentosum, Bazex–Dupré–Christol syndrome, or Oley syndrome, for instance [15]. 

**Figure 1:**
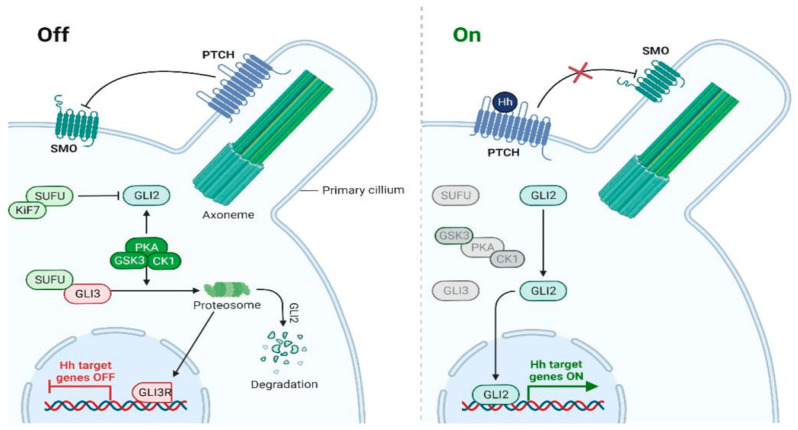
Outline of the HH signaling pathway [16]. If an Hh ligand is present, SMO is phosphorylated, and SUFU is re-activated, while GLI2 induces Hh target genes of transcription. However, if an Hh ligand is absent, GLI is phosphorylated by PKA, GSK3, and CK1, forming GLI3R (GLI repressor), thus inhibiting the Hh target genes.

Beyond inherited syndromes and recent reports, a high proportion of BCCs showed modifications in the hedgehog (HH) signaling pathway (Figure 1) [16]. The HH signaling pathway is crucial for the proper development of bodily structures such as the axial skeleton, skin, or hair [17]. Thus, inappropriate activation of HH has been linked to the development of BCC and other cancers, such as pancreatic cancer or medulloblastoma. Patched 1 (PTCH1), a multi-transmembrane protein, is the receptor for Sonic HH (shh) and inhibits Smoothened (SMO) [18]. When mutated, PTCH1 is unable to bind shh and thus inhibits SMO, causing dysregulation within the HH pathway, which in turn impacts the pathogenesis of BCC. In addition to HH, the tumor protein 53 (TP53) gene has been described as the second most frequent cause of BCC as cell cycle arrest is dysregulated [19]. 

### 3.3. Clinical Features 

BCC englobes multiple variants, including superficial, nodular, morphea-like, and basosquamous types. Amongst those, nodular BCC is the most prevalent type. BCCs are generally slow-growing, skin-colored, or have a pink/pigmented plaque or nodule. They vary in diameter from a mere few millimeters to centimeters and are prone to ulceration and spontaneous bleeding episodes. The nodular type is commonly located on the face, especially on the nose, cheeks, forehead, nasolabial folds, and eyelids [20,21,22,23,24,25].

Superficial BCCs have a color similar to the nodular type; however, they are more localized to the upper body (shoulders, back, chest). They are frequently present in multiples and can easily be confused with alternative skin conditions such as psoriasis or atopic dermatitis. Interestingly, superficial BCCs can partially transform into nodular BCCs [22]. 

The morphea-like type, also known as morpheaform BCC, is described as a sclerosing, smooth, white, or skin-colored BCC with poorly defined borders. However, they can also ulcerate within a sclerotic plaque. They tend to arise on the face and neck [26]. Due to the drastic difference between nodular and superficial BCCs, the morpheaform type of BCC is notoriously difficult to diagnose. The diagnostic challenge is particularly problematic as BCCs are more likely to metastasize and have a poorer prognosis than other BCCs [27]. 

### 3.4. Treatment 

#### 3.4.1. Vismodegib

Vismodegib has been the mainstay treatment for advanced BCCs for the last decade. While proven to be safe and efficacious in clinical trials, a recent retrospective cohort study investigated its effectiveness, safety, and utilization in Germany. The study, conducted between September 2015 and March 2019, observed 66 patients across 26 centers. Results showed a 74.2% objective response rate (ORR) and a 90.9% disease control rate (DCR). The median duration of response (DOR) was 15.9 months, with a median progression-free survival (PFS) of 19.1 months. Adverse events were reported in 95.5% of patients, but no new safety concerns arose. Overall, the study affirmed Vismodegib’s effectiveness and safety in treating laBCC, validating its use in routine clinical practice [28].

#### 3.4.2. Sonidegib

Sonidegib, approved by the Food and Drug Administration (FDA) in 2015, is an oral treatment for BCC that selectively antagonizes the SMO receptor, which is crucial for the proper function of the HH pathway. The adoption of HH signaling pathway inhibitors (HHIs) like vismodegib and sonidegib for the treatment of locally advanced BCC (laBCC) and metastatic BCC (mBCC) offered alternatives to standard surgical and radiotherapies [26]. While both drugs gained regulatory approval—vismodegib through the ERIVANCE study and sonidegib through BOLT—their comparative efficacy and safety profiles remain uncertain due to differences in study designs. Vismodegib and sonidegib differ in their pharmacokinetic behaviors; sonidegib exhibits non-concentration-dependent plasma protein binding with a greater half-life and tissue distribution, contrasting with vismodegib’s concentration-dependent binding and limited tissue distribution. The BOLT and ERIVANCE trials, while similar, employ distinct methodologies, such as modified RECIST and more strict criteria in BOLT and RECIST version 1.0 in ERIVANCE, complicating direct comparisons. After adjusting for the study differences, sonidegib demonstrated a higher ORR at 30 months compared to vismodegib at 21 months and with a few more cases of CR. Both drugs exhibited similar rates of discontinuation due to adverse events (AEs), with sonidegib showing slightly lower AE incidences but similar patterns, which included muscle spasms, alopecia, and dysgeusia, likely suggesting class-dependent effects. However, there have been no trials that directly compare these drugs (Table 1) [29].

In summary, both vismodegib and sonidegib are efficacious treatment options for BCC, although sonidedib proved to have slightly lower rates of discontinuation. Nevertheless, as aforementioned, comparisons are difficult due to differences in study designs. Further investigations are recommended to determine if one of the two treatments is superior to the other (Table 2).

#### 3.4.3. Cemipilimab

Cemiplimab gained FDA approval as a second-line therapy for adults with laBCC or mBCC. It was authorized specifically in the case of resistance to HHIs or intolerance. Before its approval in 2021, there was no second-line treatment for these conditions. BCC, characterized by high mutational burden, is considered responsive to PD-1 blockade. A recent phase two open-label, multicenter, single-arm trial investigated cemiplimab [22]. Inclusion criteria were laBCC patients unsuitable for HHI therapy due to disease progression, poor drug tolerance, or lack of improvement within a 9-month period. The study excluded patients with significant autoimmune disease, previous anti-PD-1/PD-L1 treatment, or concurrent malignancy other than BCC within 3 years. Patients received IV cemiplimab every 3 weeks for 93 weeks or until completion of treatment, disease progression, intolerable toxicity, or withdrawal. Tumor response assessments were conducted regularly, along with safety assessments to monitor adverse events, vital signs, physical examinations, and laboratory tests [30]. 

The primary goal was to determine the ORR, with secondary objectives including duration of response, progression-free survival, overall survival, and safety profile. The study enrolled 84 patients with laBCC, predominantly in the head and neck region, and observed an ORR of 31% by independent central review, with disease control achieved in 80% of patients. Median progression-free survival (MPFS) was determined to be 19 months with manageable adverse effects in 48% of patients, including immune-mediated related adverse effects, hypothyroidism, and colitis. Notably, there were no treatment-related deaths. Limitations of the study include a small sample size and a single-arm design. Moreover, exploratory biomarker analyses suggest the need for further investigation into predictive markers for response to cemiplimab [30]. 

In sum, cemiplimab is a necessary addition to the umbrella of the regiment available for BCC. Additionally, this trial showed a longer duration of treatment for a proper response in BCC, which is optimal. However, further trials with a larger sample size and inclusion of individuals with autoimmune diseases may provide a wider understanding of cemiplimab’s efficacy. 

## 4. Squamous Cell Carcinoma

### 4.1. Epidemiology 

Limited data regarding the prevalence, incidence, and mortality of cSCCs are available. However, similar to BCC, these values vary based on population. For instance, cSCC is more common in fair skin. In North America, the average incidence is 60 per 100,000 Person Years (PYs), while in Europe, it was, on average, lower, with 17.5 cases per 100,000 PYs in Norway. As exposure to sunlight varies based on latitude, it is not surprising that Australia showed a higher incidence of SS, with close to 387 cases per 100,000 PYs, with variability based on latitude within the country. While rates are increasing in Caucasian populations, they have been known to stabilize in certain areas, such as Canada [31].

Regarding mortality rates (MRs), they are equally unclear and poorly documented. A U.S.-based study recently showed a rate of 0.52 per 100,000 for keratinocyte cancer (KC). Additionally, the MR was three times higher in men than women and three times higher in fair skin when compared to black skin [32,33].

cSCC accounts for 20% of KCs; thus, this data partially reflects MRs for cSCCs [33]. However, MRs would likely increase if cancer registries were more comprehensive when recording these data. Mortality data from Germany demonstrate a 5-year relative survival rate of 94%. Norway shows slightly lower survival, with 82% (men) and 88% (women) for local disease and 64% (men) and 51% (women) for advanced disease, respectively. While these studies provide an estimate of mortality rates, it is considered insufficient and unclear. Nonetheless, SSCs are rarely fatal, particularly if detected early enough. Thus, prevention and early detection are key. This information should be emphasized via national educational prevention programs and restricted access to UV exposure from tanning beds, for instance [32,33].

### 4.2. Risk Factors

Risk Factors (RFs) for cSCC are largely similar to those of BCC. UV exposure (UVB in particular) stimulates carcinogenesis with a mutation in p53, for instance. The use of tanning beds before the age of 25 years old is a high-risk example for developing cSCC. As for BCC, UV exposure, male sex, fair skin, age, immunosuppression [34,35,36,37], inherited syndromes, glucocorticoid use, and arsenic are all RFs. Immunosuppressed patients, particularly organ transplant recipients, exhibit higher accrual rates of cSCCs than immunocompetent patients. In a retrospective cohort study, the immunocompetent group had accrual rates of (0.44 ± 0.36) versus (0.82 ± 0.95) for immunosuppressed individuals. Amongst these patients were individuals affected by psoriasis [36,38]. It is worth noting that patients treated for psoriasis with UVA or psoralen are at increased risk due to increased photosensitivity.

Actinic keratosis (AK) can be a predictor of the development of cSCC in previously unaffected individuals, although the data are hazy [38,39,40,41]. A 2-year retrospective study conducted between 2003 and 2005 demonstrated a 10% progression of AKs to cSCCs in 2 years [32]. However, recent studies show that 0–0.075% of AKs progress to cSCCs. Further investigation of their association is worth investigating, as AKs are easily removed; thus, their monitoring could prevent the development of cSCCs [41,42].

cSCCs have varying outcomes and risk of fatality based on their location and invasion. cSCCs on the head and neck generally have poorer outcomes. A 2017 retrospective cohort study investigated 36 patients with N3 (N-representing lymph node involvement) nodal head and neck cSCCs [42,43]. These patients exhibited a 5-year overall survival rate of 30%, which is significantly lower than the rates discussed above. Similarly, there is reduced survival in the case of perineural or nodal involvement, thick lesions, or marked inflammation. In summary, due to the risk of local invasion or lymphatic spread, metastases, and recurrence, early detection is crucial [43].

### 4.3. Clinical Features

CSCCs are frequently found on the face, neck, forearms, hands, and shins. Their appearance ranges from crusty, scaly lesions to ulcerations and hyperkeratosis. These ulcerations can appear like an open sore that bleeds easily. Similarly to BCC, cSCC is divided into categories: nodular, plaque, and flat. Regarding color, they span from skin-colored to erythematous [44,45]. Evidently, that color appearance is dependent on the patient’s skin tone and will look drastically different between white and black skin, for instance. The lesions of cSCC can be asymptomatic or pruritic and painful. When tender, it is crucial to investigate symptoms of perineural invasion, such as a burning sensation or paresthesia, due to an increased mortality rate [44].

### 4.4. Treatment

#### 4.4.1. Cemiplimab

cSCC is largely treated with surgery; however, a small percentage becomes metastatic or locally advanced, thus necessitating systemic therapy. cSCC presents molecular features suggesting responsiveness to systemic immune therapy, such as a high mutation burden. Cemiplimab, described in Section 3.4.3 showed promising results in the treatment of laSCC and mSCC in a 2018 clinical trial [32,36,46].

The study investigates cemiplimab’s efficacy in the treatment of laSCC and mSCC, which previously lacked systemic treatment options. The higher mutation burden of this tumor has predicted its responsiveness to the immune checkpoint blockers. Moreover, the increased risk of SCC in the immunocompromised has led to an investigation of the role of immune surveillance in this disease. The results of the study were reported by measuring the objective response rate (ORR) in phase 1 and 2 studies in a cohort that included patients with laSCC and mSCC, respectively. Phase 2 trials for laSCC are ongoing. Phase 1 and 2 trials demonstrated robust efficacy, with consistent response rates (RR) of 50% and 47%. Most of the side effects did not exceed grade 2 and were found to be PD-1 class related. The most common side effects were found to be diarrhea, fatigue, and rash. Nevertheless, a small percentage of participants discontinued the treatment because of the severe adverse effects. In summary, cemiplimab’s efficacy was highlighted in the treatment of cSCC, which aligns with existing evidence on immune checkpoint blockade efficacy for hypermutated cancers (Table 2) [46].

**Figure 2:**
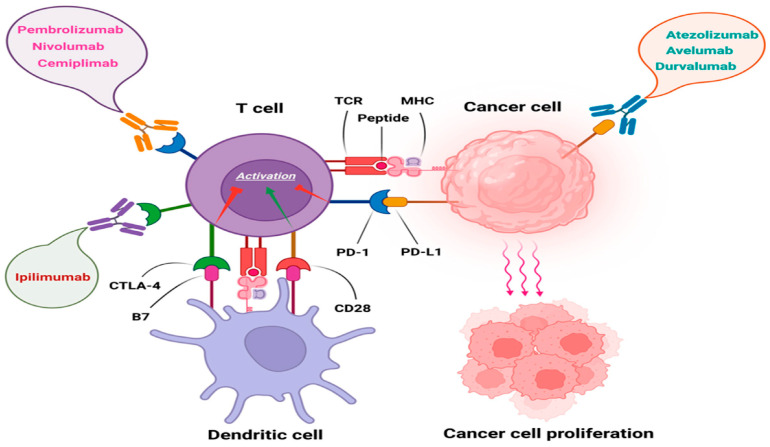
Immune checkpoint inhibitors approved by the FDA. Pembrolizumab, Nivolumab, and Cemiplimab as anti-PD-1 antibodies, Ipilimumab as an anti-CTLA-4 antibody, as well as Atezolizumab, Avelumab, and Durvalumab as anti-PD-L1 antibodies [48].

**Figure 3:**
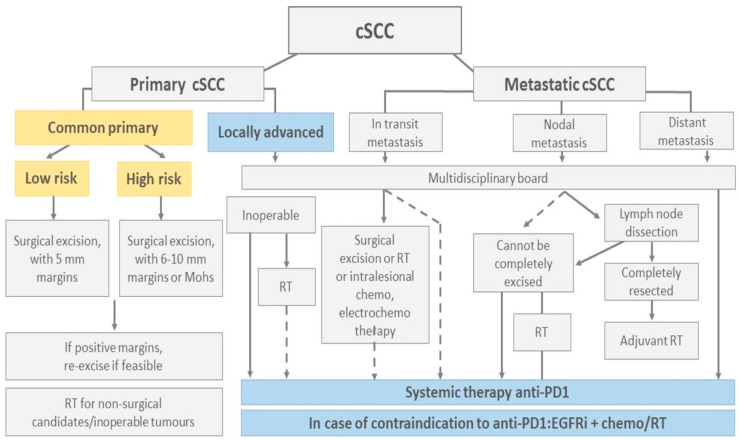
Outline of the European guidelines for the main therapeutic options for cSCC [51,52]. This review focuses on systemic treatment for locally advanced and metastatic cSCC, which are considered if the tumor is inoperable or if surgery is insufficient. Within systemic treatment, there are different options, which are described in Table 2 above.

Another pivotal, open-label, phase 2, single-arm trial investigating the efficacy of cemiplimab in the treatment of laSCC was published in 2020 [30]. Over the enrollment period from 14 June 2016 to 25 April 2018, a cohort of 78 patients received cemiplimab treatment intravenously infused at 3 mg/kg every 2 weeks for up to 96 weeks, with tumor assessments performed every 8 weeks. Objective responses were evaluated based on the proportion of patients exhibiting complete or partial responses based on RECIST 1.1 and WHO radiologic and photographic criteria, with independent and investigator assessments noting responses in 44% and 53% of patients, respectively. The median follow-up duration was 9.3 months, during which durable responses were observed, with the longest response ongoing at 24.2 months. Ten (13%) patients demonstrated a complete response, and twenty-four (31%) showed a partial response. Adverse events, notably grade 3–4 treatment-emergent adverse events, occurred in 33 (44%) patients, with common occurrences including hypertension, pneumonia, hepatitis, and elevated liver enzymes. One death resulted from aspiration pneumonia. Immunohistochemistry analysis of tumors did not demonstrate a correlation between cemiplimab’s clinical activity and baseline PD-L1 TPS, and the range of mutational burden of tumors was inconsistent in both responders and non-responders, highlighting the need for further studies to evaluate tumor markers in SCC that might better predict cemiplimab’s benefit to patients. Limitations of the investigation encompass the single-arm design, small number of patients, absence of long-term follow-up, and the use of a non-survival primary endpoint. The data reported in the study further support the approval of cemiplimab by the FDA in September 2018 and by the European Commission in June 2019 for the treatment of laSCC and mSCC, which are not suitable for curative surgery or radiation. Moreover, the study investigated a dose of 3 mg/kg every two weeks, and the FDA-approved dose constitutes 350 mg every three weeks and is being examined separately (Table 3) (Figure 2 and Figure 3) [47].

#### 4.4.2. Pembrolizumab

Similarly to cemiplimab, other PD-1 inhibitors are undergoing trials to determine their efficacy and safety in treating cSCC. A 2021 study investigated patients with laSCC or recurrent/metastatic (R/M) cutaneous squamous cell carcinoma (cSCC) [49]. The KEYNOTE-629 trial was a global, open-label, nonrandomized, phase 2 study conducted from 29 November 2017 to 25 September 2019. A total of 159 patients (laSCC: *n* = 54; R/M SCC cohort: *n* = 105) were treated with IV pembrolizumab 200 mg every 3 weeks for up to 35 cycles. The primary endpoint was ORR, with secondary endpoints including duration of response, disease control rate, progression-free survival, overall survival, and safety. Results showed that pembrolizumab demonstrated clinically meaningful and durable antitumor activity in both cohorts, with manageable safety profiles. In the laSCC cohort, ORR was 50.0%, with the median duration of response not yet reached; in the R/M cohort, ORR was 35.2%. Median progression-free survival was not reached in the laSCC cohort and was 5.7 months in the R/M cohort. Median OS was not reached in the laSCC cohort and was 23.8 months in the R/M cohort. Treatment-related adverse events affected 69.2% of patients, with grade 3–5 events developing in 11.9%. There were two documented treatment-related deaths. While clinical activity was evident across the entire patient population irrespective of PD-L1 status, there was a significant trend of improved response in patients with higher levels of PD-L1 expression. Limitations of this study include the single-arm design and small first-line treatment subgroup in the R/M cohort. Pembrolizumab’s efficacy and safety were found sufficient for it to be established as a significant treatment option for advanced cSCC, with potential benefits in the adjuvant setting following surgery and radiation therapy, as ongoing phase III studies. Pembrolizumab received FDA approval for LA cSCC in July 2021 based on these findings (Table 2) (Figure 2 and Figure 3) [49].

#### 4.4.3. Nivolumab

Outside of cemiplimab and pembrolizumab, Nivolumab has been investigated as a treatment for cSCC (Table 2). Its efficacy was researched in systemic treatment-naive patients with a CSCC in this phase 2 open-label trial, investigated in systemic treatment-naive patients [49]. The study enrolled 24 patients with a median age of 74 years. Nivolumab was administered intravenously at 3 mg/kg every 2 weeks until disease progression, intolerable toxicity, or completion of 12 months of treatment. Assessments included tumor biopsies, radiographic evaluations using RECIST 1.1 criteria, and monitoring of treatment-related adversities. The primary endpoint was the best objective response rate at 24 weeks, with secondary endpoints including progression-free survival and overall survival. At a median follow-up of 17.6 months, the ORR was 58.3%, with median progression-free survival and overall survival estimated at 12.7 and 20.7 months, respectively. Notably, adverse events occurred in 87.5% of patients, with 25% experiencing adverse effects of grade 3 and above events, leading to one treatment discontinuation. The most common treatment-related side effects were hypothyroidism (33%), pruritus (33%), fatigue (29%), lymphopenia (29%), arthralgia (29%), and rash (25%). Overall, treatment-related side effects of any grade occurred in 87.5% of patients, with the majority being grade 1 or 2 events (62.5%). There were no documented treatment-related deaths. Limitations of the study included its small sample size, lack of a comparative arm, and potential biases inherent to nonrandomized trial designs. Despite these limitations, nivolumab demonstrated robust antitumor activity and manageable side effects in systemic treatment-naive patients with a CSCC, supporting its use as a standard treatment option for this population (Figure 2 and Figure 3) [50].

## 5. Discussion

Skin cancers possess a heightened immunogenicity stemming from the presence of tumor-associated antigens, mutations, and/or expression of viral genes. Recent observations indicate a global surge in skin cancer rates, primarily attributed to several key factors, including climatic shifts, fair skin complexion, prolonged exposure to sunlight, advanced ages, and low social conditions [1,5,9,52,53,54,55,56,57,58,59,60].

NMSC stems from epidermal cells and exhibits typical epidemiological patterns, such as a higher occurrence among Caucasian populations. Although the development of BCC and cSCC can be due to exposure to physical carcinogens, ultraviolet radiation (UVR) stands out as the predominant risk factor. UVR can directly induce the malignant transformation of precursor cells [55,56,57,58,59,60,61,62,63,64,65]. Additional risk factors for the onset of BCC and cSCC encompass various aspects, including pre-existing conditions and their respective treatments (e.g., psoriasis), targeted therapies utilized for other cancer types, notably melanoma, immune suppression induced by medications in transplant recipients, and prolonged exposure to human papillomavirus. Additionally, multiple studies have indicated that individuals with lower socioeconomic status are more prone to developing non-melanoma skin cancers (NMSCs) [7,18,55,56,57,58,59,60,62,63,64,65,66,67,68,69].

Advancements in the knowledge of the pathogenesis of BCCs and cSCCs have paved the way for the development of innovative therapies, significantly impacting the survival and quality of life for numerous patients. Notably, insights into the genetic makeup of BCCs have underscored the critical role of the Hedgehog pathway in driving cancer cell proliferation, with targeted agents effectively inhibiting this pathway’s activity [52]. Additionally, new genetic defects have been identified, opening avenues for alternative treatment approaches such as PD-1 inhibitors like cemiplimab or pembrolizumab as an option for first-line treatment for individuals diagnosed with locally advanced cSCC or patients who are ineligible for radical surgery or radiotherapy, or for patients who are diagnosed upfront with metastatic cSCC [1,5,9,18,53,54,55,56,57,58,59,60,61,62,63,64,65,66,67,68,69,70].

In the context of cSCC, the immune system emerges as a pivotal player in disease pathogenesis, with preclinical models offering valuable insights into immune cell alterations that influence skin cancer biology [69,70,71,72,73,74,75]. However, cSCC development is likely multifactorial, with a complex interplay between genetic and environmental factors contributing to keratinocyte malignant transformation. Comparative studies between premalignant and malignant skin tissues have uncovered differences in proteomic, genomic, and immunological profiles associated with cancer development [17,55,56,57,58,59,60,61,62,63,64,65,67,68,69,70,71,72].

Exposure of the skin to UVR initiates various responses at the local level, driven by chemical, hormonal, immune, and neural signals influenced by the specific chromophores present and the depth of UV penetration into different skin layers. These signals trigger a cascade of electrical, chemical, and biological responses transmitted to the brain, endocrine system, immune system, and other vital organs, collectively orchestrating the body’s equilibrium. Consequently, there are subsequent alterations in keratinocyte cytokine signaling, leading to immunosuppressive effects on T-cell activities, ultimately contributing to an increased risk of cancer development [73,74,75,76,77,78,79,80,81].

Furthermore, UV radiation, a well-known carcinogen implicated in the formation of BCC and cSCC, also stimulates the production of vitamin D in the skin. Vitamin D3, a prohormone, is enzymatically activated via a canonical pathway by sequential hydroxylation at C25 and C1alpha. Additionally, it is activated in an alternative pathway by CYP11A1 [67,68,69,70,71,72,73,74,75,76,77,78]. Vitamin D exhibits not only photoprotective properties but also demonstrates anti-cancer activity, offering additional defense mechanisms against UV-induced malignancies [80,81,82,83,84,85,86,87,88].

Moreover, mechanisms of positive UVR modulation of the immune system are complex and extend beyond vitamin D regulation. A study has demonstrated that UVR has beneficial immunomodulatory effects on multiple sclerosis in the experimental autoimmune encephalomyelitis model that was found to be independent of vitamin D production. UVR can be immunosuppressive by stimulating the central and cutaneous hypothalamic–pituitary–adrenal (cHPA) axis, leading to the production of various peptides and steroids such as α-MSH, β-endorphin, ACTH, and corticosterone. This leads to systemic immunosuppression that involves both neural and humoral mechanisms, highlighting the complex regulatory role of UVR in maintaining tissue homeostasis and immune function [85,86,87,88,89].

Additionally, investigations into microenvironmental defects in NMSCs have highlighted dynamic interactions between malignant cells and components regulating both innate and adaptive immune responses. Understanding these processes has revolutionized the approach to treating metastatic cSCC, offering novel therapeutic options that are also being explored in the management of BCC [19,90,91,92].

It should be noted that numerous clinical trials are currently underway, exploring treatments for advanced and metastatic non-melanoma skin cancers, cSCC, and BCC (Table 4).

## 6. Conclusions

Recent advancements in therapies, such as immunotherapies and Hedgehog inhibitors, have transformed treatment options in oncology, significantly benefiting patients with common skin cancers like BCC, cSCC, and melanoma, leading to improved survival rates. However, despite significant progress, a substantial number of patients do not achieve a cure with these treatments. Therefore, additional research is imperative to further enhance treatment outcomes.

## Figures and Tables

**Table 1:** Summary table of the BOLT clinical trial investigating the use of Sonidegib.

Measure	laBCC 200 mg (*n* = 66)	laBCC 800 mg (*n* = 128)	mBCC 200 mg (*n* = 13)	mBCC 800 mg (*n* = 23)
**Efficacy Outcomes**				
ORR, % (95% CI)	56 (43–68)	46.1 (37.2–55.1)	8 (0.2–36)	17 (5–39)
CR, % (95% CI)	5 (0.9–13)	1.6 (0.2–5.5)	0 (0–25)	0 (0–15)
Adverse Effects				
Muscle spasms	43 (54%)	104 (69.3%)	-	-
Alopecia (Grade ≤ 2)	39 (49%)	87 (58.0%)	-	-
Elevated CK (Grade 3–4)	5 (6%)	20 (13.3%)	-	-
Elevated lipase (Grade 3–4)	5 (6%)	-	-	-
Pneumonia (Serious)	2 (3%)	-	-	-
Elevated CK (Serious)	-	6 (4.0%)	-	-

Abbreviations: Locally advanced basal cell carcinoma (laBCC), metastatic basal cell carcinoma (mBCC), Objective response rate (ORR), Creatine Kinase (CK), Control rate (CR).

**Table 2:** Summary table of available hedgehog inhibitors in the treatment of basal cell carcinoma, including their FDA approval, Clinical trial results, and common adverse effects.

Treatment	Indication	FDA Approval	Clinical Trial Results	Common Adverse Effects
Vismodegib	1st line	2012	ORR: 74.2% DCR: 90.9% MPFS: 15.9 months	Muscle spasms Alopecia Dysgeusia Fatigue Abnormal weight loss Taste disorder
Sonidegib	1st line	2015	Variable based on dose—See Table 1	Muscle spasms Alopecia Dysgeusia

Abbreviations: Objective response rate (ORR), Median progression-free survival (MPFS), Overall survival (OS), Disease control rate (DCR).

**Table 3:** Summary of the treatment options available for BCC and cSCC. This table includes FDA approval, clinical trial results, and common adverse effects. Indications refer to 1st line or 2nd line for locally advanced and metastatic BCC and cSCC that have not been treatable with surgical excision.

Treatment	Indication	FDA Approval	Clinical Trial Results		Common Adverse Effects
Vismodegib	1st line	2012	ORR: 74.2% DCR: 90.9% MPFS: 15.9 months		Muscle spasms Alopecia Dysgeusia Fatigue Abnormal weight loss Taste disorder
Sonidegib	1st line	2015	Variable based on dose—See Table 1		Muscle spasms Alopecia Dysgeusia
Cemiplimab	1st line	2018 (advanced/metastatic cSCC)	laSCC	mSCC	Diarrhea Fatigue Rash Hypertension Pneumonia Hepatitis
			RR: 50%	RR: 47%	
Pembrolizumab	1st line	2020	laSCC	R/T	Pruritus Fatigue Asthenia Rash Diarrhea Hypothyroidism Nausea
			ORR: 50% MPFS: not yet reached OS: not reached	ORR: 35.2% MPFS: 5.7 months OS: 23.8 months	
**Nivolumab**	Off label use	Not yet approved for cSCC (Off-label use)	at 17.6 months ORR: 58.3%		Hypothyroidism Pruritus Fatigue Lymphopenia Arthralgia Rash

Abbreviations: Cutaneous Squamous cell carcinoma (cSCC), Locally advanced squamous cell carcinoma (laSCC), metastatic squamous cell carcinoma (mSCC), Objective response rate (ORR), Median progression-free survival (MPFS), Overall survival (OS), Response rate (RR), Disease control rate (DCR).

**Table 4:** Summary table of ongoing clinical trials investigating treatment for advanced and metastatic non-melanoma skin cancers.

NCT Number	Phase	Study Title	Conditions	Interventions	Primary and Secondary Endpoints
NCT05267626	II	Study of AU-007, A Monoclonal Antibody That Binds to IL-2 and Inhibits IL-2Rα Binding, in Patients With Unresectable Locally Advanced or Metastatic Cancer	Metastatic cancer (non-melanoma skin cancer)	DRUG: AU-007|DRUG: Aldesleukin	Safety
NCT03775525	I/Ib	Study Evaluating GZ17-6.02 in Patients With Advanced Solid Tumors or in Combination With Capecitabine in Metastatic Hormone Receptor Positive Breast Cancer	cSCC and BCC	DRUG: GZ17-6.02|DRUG: Capecitabine	DLTs
NCT05592626	I/II	A Study of a Selective T Cell Receptor (TCR) Targeting, Bifunctional Antibody-fusion Molecule STAR0602 in Participants With Advanced Solid Tumors	Metastatic Carcinomas	DRUG: STAR0602	Dose Escalation, ORR, DOR
NCT04913220	I/II	A Study of SAR444245 Combined With Cemiplimab for the Treatment of Participants With Various Advanced Skin Cancers (Pegathor Skin 201)	cSCC	DRUG: THOR-707|DRUG: Cemiplimab	ORR, DLTs
NCT05086692	I/II	A Beta-only IL-2 ImmunoTherapY Study	BCC CsCC MCC	DRUG: MDNA11|DRUG: Pembrolizumab	DLTs
NCT04812535	II	Non-comparative Study of IFX-1 Alone or IFX-1 + Pembrolizumab in Patients With Locally Advanced or Metastatic cSCC.	cSCC	DRUG: IFX-1|DRUG: IFX-1 + pembrolizumab combination therapy	ORR, DLTs
NCT06041802	II	A Study of MK-3475A (Pembrolizumab Formulated With MK-5180) in Japanese Participants With Recurrent or Metastatic Cutaneous Squamous Cell Carcinoma (R/M cSCC) or Locally Advanced (LA) Unresectable cSCC (MK-3475A-E39)	cSCC	BIOLOGICAL: MK-3475A	ORR, DOR
NCT02955290	I/II	CIMAvax Vaccine, Nivolumab, and Pembrolizumab in Treating Patients With Advanced Non-small Cell Lung Cancer or Squamous Head and Neck Cancer	cSCC	OTHER: Laboratory Biomarker Analysis|BIOLOGICAL: Nivolumab|BIOLOGICAL: Pembrolizumab|BIOLOGICAL: Recombinant Human EGF-rP64K/Montanide ISA 51 Vaccine	ORR, DOR, DCR, OS
NCT04799054	I/II	A Study of TransCon TLR7/8 Agonist With or Without Pembrolizumab in Patients With Advanced or Metastatic Solid Tumors	cSCC	DRUG: TransCon TLR7/8 Agonist|DRUG: Pembrolizumab	Safety, ORR
NCT03944941	II	Avelumab With or Without Cetuximab in Treating Patients With Advanced Skin Squamous Cell Cancer	cSCC	DRUG: Avelumab|DRUG: Cetuximab	PFS, ORR, OS
NCT05620134	I/II	Study of JK08 in Patients With Unresectable Locally Advanced or Metastatic Cancer	cSCC	DRUG: JK08|DRUG: Pembrolizumab	DLTs, Safety
NCT05238363	II	HLX07 in Locally Advanced or Metastatic Cutaneous Squamous Cell Carcinoma (CSCC)	cSCC	DRUG: HLX07	PFS, ORR, OS, DOR
NCT06270706	I	PLN-101095 in Adults With Advanced or Metastatic Solid Tumors	Metastatic Solid Tumors	DRUG: PLN-101095|DRUG: Pembrolizumab	DLTs
NCT02268747	II	Dacomitinib Treatment of Skin Squamous Cell Cancer	cSCC	DRUG: Dacomitinib	DCR, OS, PFS
NCT02721732	II	Pembrolizumab in Treating Patients With Rare Tumors That Cannot Be Removed by Surgery or Are Metastatic	cSCC	OTHER: Laboratory Biomarker Analysis|BIOLOGICAL: Pembrolizumab|OTHER: Questionnaire Administration	ORR, OS, DCR, DOR
NCT02964559	II	Pembrolizumab in Patients With Locally Advanced or Metastatic Skin Cancer	cSCC	BIOLOGICAL: Pembrolizumab	PFS, OS
NCT05970497	I	A Study Assessing KB707 for the Treatment of Locally Advanced or Metastatic Solid Tumors	cSCC BCC	BIOLOGICAL: KB707	DLTs, Safety, ORR

Abbreviations: Basal Cell Carcinoma (BCC), Cutaneous Squamous Cell Carcinoma (cSCC), Merkel Cell Carcinoma (MCC), Dose-limiting toxicities (DLTs), Duration of Responses (DOR), Objective response rate (ORR), Disease Control Rate (DCR), Overall Survival (OS), Progression-free survival (PFS).

## Data Availability

Data are contained within the article or are available from the authors upon reasonable request.
